# Using a composite adherence tool to assess ART response and risk factors of poor adherence in pregnant and breastfeeding HIV-positive Cameroonian women at 6 and 12 months after initiating option B+

**DOI:** 10.1186/s12884-018-2058-9

**Published:** 2018-10-25

**Authors:** Pascal N. Atanga, Harrison T. Ndetan, Peter N. Fon, Henry D. Meriki, Tih P. Muffih, Eric A. Achidi, Michael Hoelscher, Arne Kroidl

**Affiliations:** 1 Cameroon Baptist Convention Health Service (CBCHS), P. O. Box 152, Tiko, Health Services Complex, Mutengene, South West Region Cameroon; 2 Department of Public Health and Hygiene, Faculty of Health Sciences, University of Buea, P.O. Box 63, Buea, Cameroon; 3 Centre for International Health (CIH), University of Munich (LMU), Munich, Germany; 4 Department of Epidemiology and Biostatistics, University Texas Health Northeast, School of Community and Rural Health, Tyler, USA; 5 Department of Biostatistics and Epidemiology, School of Public Health, University of North Texas Health Science Center, Fort Worth, TX USA; 6 Faculty of Science, University of Buea, P.O. Box 63, Buea, Cameroon; 7 Department of Microbiology and Parasitology, Faculty of Sciences, University of Buea, P.O. Box 63, Buea, Cameroon; 8 Laboratory Department, Regional Hospital Buea, Buea, Cameroon; 9 Division of Infectious Diseases and Tropical Medicine, Medical Centre of the University of Munich (LMU), Munich, Germany; 10 German Centre for Infection Research (DZIF), Partner Site Munich, Munich, Germany

**Keywords:** Option B +, Adherence, Viral load, Risk factors, Cameroon

## Abstract

**Background:**

Antiretroviral therapy (ART) adherence in preventing HIV mother-to-child transmission in association with virological suppression and risk factors of low adherence in the Cameroon’s Option B+ programme are poorly understood. We used a composite adherence score (CAS) to determine adherence and risk factors of poor adherence in association with virological treatment response in HIV-positive pregnant and breastfeeding women who remained in care at 6 and 12 months after initiating ART.

**Methods:**

We prospectively enrolled 268 women after ART initiation between October 2013 and December 2015 from five facilities within the Kumba health district. Adherence at 6 and 12 months were measured using a CAS comprising of a 6-month medication refill record review, a four-item self-reported questionnaires and a 30-day visual analogue scale. Adherence was defined as the sum scores of the three measures and classified as high, moderate and low. Measured adherence levels were compared to virological suppression rates at month 12 and risk factors of poor adherence were determined.

**Results:**

At 6 and 12 months, 217 (81.0%) and 185 (69.0%) women were available for adherence evaluation. Respectively. Of those, 128 (59.0%) and 68 (31.4%) had high or moderate adherence as per the CAS tool at month 6, and 116 (62.7%) and 48 (24.9%) at month 12, respectively. Viral loads were assessed in 165 women at months 12, and 92.7% had viral suppression (< 1000 copies/mL). Viral suppression was seen in 100% of women with high, 89.5% with moderate, and 52.9% with low adherence using the CAS tool. Virological treatment failure was significantly associated with low adherence [OR 7.6, (95%CI, 1.8–30.8)]. Risk factors for low adherence were younger age [aOR 3.8, (95%CI, 1.4–10.6)], primary as compared to higher levels of education [aOR 2.7, (95%CI, 1.4–5.2)] and employment in the informal sector compared to unemployment [aOR 1.9, (95%CI,1.0–3.6)].

**Conclusions:**

During the first year of Option B+ implementation in Cameroon our novel CAS adherence tool was feasible, and useful to discriminate ART adherence levels which correlated with viral suppression. Younger age, less educated and informal sector employed women may need more attention for optimal adherence to reduce the risk of virological failure.

**Electronic supplementary material:**

The online version of this article (10.1186/s12884-018-2058-9) contains supplementary material, which is available to authorized users.

## Background

Adherence to antiretroviral therapy (ART) for HIV infection is essential for plasma viral load suppression. This is key to treatment success as it reduces both morbidity and mortality but most importantly improves the quality of life and reduces drug resistance development [1–3]. Non-adherence is the most important factor that leads to viral resistance [4]. As test and treat is being adopted worldwide including Option B+ procedures for newly diagnosed HIV-positive pregnant and breastfeeding women, additional adherence and retention support are needed as most of the patients initiating ART present with asymptomatic HIV infection [5, 6]. Since the introduction of Option B+ in Malawi in 2011, ART uptake has significantly improved for this target group but there are still concerns that women with asymptomatic HIV infection may not be adequately retained or poorly adhere to treatment [7–9]. Recent studies since the introduction of Option B+ have focused on retention in care [8, 10, 11] and very little data is available assessing patients’ adherence to ART. Evidence also shows that retention, which is often used as a surrogate for adherence, may fail to account for the pattern of clinic attendance and drug taking behaviour [12]. The few studies evaluating adherence to Option B+ used a single adherence measure such as self-report or pharmacy refill which both have been shown to overestimate adherence [13, 14]. Overestimated adherence could result in patient misclassification and lead to inaccurate targeting of adherence-improving interventions or delays in addressing adherence problems. Self-reported adherence is subjective and its reliability drops overtime as patients get acquainted with the assessment tool used [15]. Pharmacy refills and pill counts on their part, though objective and not easily affected by recall and social desirability biases, may result in ‘pill-dumping’, thus overestimating adherence [16, 17]. Viral load (VL) suppression data can provide additional information to improve adherence assessment conducted using any subjective measure, though it could also be affected by pre-treatment HIV drug resistance [18]. To optimise the Option B+ programme and reduce the risk of treatment failure, timely and adequate assessment of adherence in HIV-positive pregnant and breastfeeding women with a simple but robust tool is critical in resource limited settings where second line medication options are limited and third line regimens are virtually non-existent or are still very expensive [2, 5]. Sub-optimal adherence needs to be identified and addressed early prior to treatment failure and the development of viral drug resistance. This is possible only if we understand the risk factors of poor adherence and those enablers of proper medication taking behaviour. These factors have been studied in other settings but are poorly understood in Cameroon [19, 20]. In order to meet the UNAIDS 90–90-90 objective by 2020 [21], treatment programmes should not only focus on HIV testing, ART initiation and retention in care, but also on adherence to lifelong ART which is key to reaching the 3rd 90. However, notwithstanding the importance of near perfect adherence on viral suppression, recent research findings are showing that newer ART regimens may only require moderate adherence levels to achieve viral suppression [22–24].

In our previous analysis of this cohort of pregnant or breastfeeding women, we observed good uptake of HIV testing and counselling, ART and retention in care, which however, declined over time. We analysed treatment discontinuation taking into account women lost to follow up, transferred out to other ART clinics, intentionally stopped medication or died. Discontinuation from Option B+ was highest at small sites with a high staff turnover [8]. Based on those findings, we now specifically evaluated information on women who remained in care with respect to ART adherence in association with viral suppression using a multiple method tool. We further investigated risk factors associated with poor adherence alongside enablers of proper drug taking behaviour.

## Methods

### Study design and settings

This prospective cohort study was carried out between October, 2013 and December 2015 at five health facilities located within the Kumba Health District, South West Region, Cameroon, which all provide integrated maternal, neonatal, child health and prevention of mother to child transmission (PMTCT) services. These sites were among the ten facilities that piloted Option B+ in the region from October, 2013. They receive well over 90% of all pregnant women seeking antenatal care (ANC) in the health district, and also had their staff trained in Option B+ procedures and task shifting as part of the pilot project. Task shifting allows midwives and nurses to prescribe ART and follow-up the mother baby pair [25].

### Study participants, procedures, outcomes and definitions

The study participants and procedures were previously described [8]. For the current analysis, which focused on adherence using a composite adherence score and viral suppression, we included women who remained in care at six and twelve months after ART initiation. In brief, ART was offered as a once daily regimen with a fixed dose combination of tenofovir, lamivudine and efavirenz regardless of clinical or immunological status in accordance with WHO and national guidelines [5, 26]. ART follow-up cards were opened for each ART initiating client from which information on age, date and timing of ART initiation, previous ART history, WHO staging, CD4 T-cell counts, level of education, profession, religious affiliation, and marital status was documented. Adherence and VL information were completed on subsequent follow-up visits when available. ART visits were scheduled monthly in a way that provided at least 4 days buffer supply of medications. Visits were managed at ANC or infant welfare clinics (IWC) post-delivery by trained nurses or midwives. During each refill visit, women received routine information and support on adherence, and every 6 months each women underwent a comprehensive adherence assessment. Data extraction and interviews were conducted by nurses and midwives who were trained as study staff. VL testing was performed 12 months after ART initiation when VL became routine for patient monitoring using the Real Ti*m*e HIV-1 *m*2000™ System (Abbott Laboratories, Illinois, USA). VL samples were not collected if either the woman missed her clinic visit on the day of sample collection or had multimonth refills during a previous visit. Virological suppression was defined as any value < 1000 copies/mL on a single measurement according to WHO and national guidelines [4, 26]. Women with low replicating viraemia (40 and 999 copies/mL) were subjected to re-enforced adherence counselling, those with suspected virological treatment failure (VL ≥1000 copies/mL) also received re-enforced adherence support and repeated VL testing within 3 months if adherence was judged satisfactory. During re-enforced adherence, individual adherence challenges were identified by psychosocial support staffs who then worked with individual clients until viral load retesting after adherence was judged satisfactory.

The primary study outcome was ART adherence at six and twelve months after Option B+ initiation using a validated multi-method tool adapted from South Africa [27]. We included medication refill record review (ARV pick-up appointments) as ARVs in Cameroon are sourced and distributed by a sole body through approved ARV treatment centres and Option B+ sites free of charge. Adherence was assessed using a six monthly medication refill record review, a four-item self-reported adherence questionnaire, and a thirty days visual analogue scale (VAS).

**Fig. 1:**
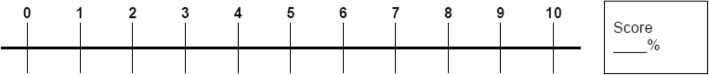
Visual Analogue Scale (VAS)

Medication refill records for each woman over the past 6 months were reviewed by the study staff to track the total number of refills effected using the patient ART cohort register. A face-to-face interview was then administered, prefaced by a normalizing language in order to reduce social desirability bias [28, 29]. The interviewer then administered the four closed ended questions formulated such that the right answer was a no to reduce the “white coat effect” [27]. The four questions used to evaluate self-reported adherence were (i) “*Do you sometimes find it difficult to remember to take your medicine?”*; (ii) *“When you feel better, do you sometimes stop taking your medicine?”*; (iii) *“Thinking back over the past 4 days, have you missed any of your doses?”*; and (iv) *“Sometimes if you feel worse when you take the medicine, do you stop taking it?”* [27]*.* Two additional questions were administered to probe for reasons of missing doses and enhancers of drug taking behaviour as follows; (i) “*What causes you to miss some doses of your drug?”, and (ii) “What helps you remember to take your drugs?”* Women were finally asked to estimate adherence on a linear VAS, (Fig. 1), which included a score ranging from zero (no adherence) to ten (optimal adherence) over the last 30 days [30, 31].

Adherence was scored separately for each adherence measure with a score on 3 indicating high, 2 moderate and 1 low adherence (Table 1). Overall adherence was estimated by creating a composite adherence score (CAS), which included the sum of the scores from each of the three measures and classified adherence as high, moderate or low. To avoid overweighting, once adherence was scored as poor on pharmacy refill the CAS was low irrespective of the scores on the questionnaire and the VAS.

**Table 1:** Interpretation of the single adherence measures and the composite adherence score (CAS)

Single adherence tool*	Medication refill	Had 6 refills (3)	Missed 1 refill (2)					Missed 2 or more refills (1)			
	Self-report questionnaire	No to all 4 questions (3)	Yes to 1 question (2)					Yes to 2 or more questions (1)			
	VAS	90% or more (3)	80% to less than 90% (2)					Less than 80% (1)			
Composite adherence tool	Medication refill score	3	3	3	3	3	2	3	2	2	1
	Self-report questionnaire score	3	3	3	2	2	2	1	2	1	1
	VAS score	3	2	1	2	1	2	1	1	1	1
	CAS	9	8	7	7	6	6	5	5	4	3
	Overall adherence	High	Moderate					Low			

*CAS* composite adherence score, *VAS* visual analogue scale; Single adherence scores: 3 = high adherence, 2 = moderate adherence, 1 = low adherence;

Secondary outcomes included risk factors of poor adherence, reasons for missing medications, reminders commonly used to enhance adherence, and the correlation between different adherence tools and the CAS. In assessing the risk factors associated with poor adherence, all women who had discontinued treatment at or before 12 months after ART initiation were considered as the worst case of adherence and were included into the low adherence category.

### Statistical analysis

Data were collected on study specific case report forms, entered into an Excel spread sheet and corrected for inconsistency before extraction for analysis. Descriptive statistics were used to examine the baseline socio-demographic, laboratory and clinical characteristics of the study participants at ART initiation. Binary logistic regression was used to assess the association between adherence scores by different adherence tools and viral suppression, as well as to assess the risk factors for poor adherence. Unadjusted odd ratios (OR) and 95% confidence intervals (CI) were reported. For risk factor, variables with significant or marginal association (*p* <  0.100) and those previously reported to be associated with poor adherence were included into the multivariate analyses reporting the adjusted odd ratios (aOR) for poor adherence. An alpha level of < 0.05 was set to define significance. Spearman correlation was used to compare the performance of the different adherence measures with each other and with the CAS. Data were analysed using the IBM Statistical Package for Social Sciences (version 21, IBM SPSS Inc., Chicago IL, USA).

## Results

### Baseline characteristics of the study participants

Of the 268 women starting Option B+, 253 (94.4%) initiated ART prior to labour/delivery and 15 (5.6%) during breastfeeding. Patients’ baseline socio-demographic, HIV status, clinical and laboratory characteristics are provided in Table 2. In brief, the median age at ART initiation was 27 (IQR 24–31) years, a minimum of primary level of education was attained in 263 (98.1%) and 105 (39.2%) were unemployed. At ART initiation the median CD4+ T-cell count was 376 cells/mL, (IQR 244–544.8), 234 (87.3%) were ART naïve, whereas 4 (1.5%) had been exposed to ARV during previous pregnancies. In addition, 30 (11.2%) women already received Option A during their current pregnancy and were switched to Option B+ triple regimens.

**Table 2:** Baseline socio-demographic, clinical and laboratory characteristics of women who started triple ART for PMTCT Option B+ in Kumba Health District, South West Region, Cameroon between October 2013 and December 2014

Variables	Description	*N* = 268
Age, years	Median (IQR)	27 (24–31)
Age groups, years	15–24	76 (28.4)
	25–34	156 (58.2)
	35 and above	36 (13.4)
Educational Level	None	5 (1.9)
	Completed primary	147 (54.8)
	Completed secondary and above	116 (43.3)
Marital Status	Single	60 (22.4)
	Married	171 (63.8)
	Others (divorced, widow)	37 (13.8)
Religious affiliation	Catholic	59 (22.0)
	Presbyterian	88 (32.8)
	Baptist	16 (6.0)
	Pentecostals	100 (37.3)
	Muslim	5 (1.9)
Occupation	Unemployed	105 (39.2)
	Employed (formal public sector)	14 (5.2)
	Employed (informal sector) or others	149 (55.6)
HIV status	Known HIV positive, not on ART	34 (12.7)
	New HIV diagnosis	234 (87.3)
ART status	ART naive	234 (87.3)
	Not on ART, but previously exposed	4 (1.5)
	Currently on Option A	30 (11.2)
Timing of ART initiation	After delivery	15 (5.6)
	Prior to labour/delivery	253 (94.4)
CD4 cell count at ART initiation, cells/μL	Median (IQR)	376 (244–544.8)
CD4 cell count groups	> 350 cells/μL	153 (57.1)
	≤350 cells/μL	115 (42.9)
WHO stage	WHO stage 1	226 (84.3)
	WHO stage 2	35 (13.1)
	WHO stage 3	7 (2.6)
	WHO stage 4	0 (0)

Data are in numbers and percentages [n (%)] or for continuous variables in median and Interquartile range (IQR)

### Adherence measurements and the performance of the different tools

After six and twelve months 217 (81.0%) and 185 (69.0%) of women were still available for adherence analysis, respectively. Adherence levels determined by CAS for women retained in care at months 6 and 12 were high in 128 (59.0%) and 116 (62.7%) cases, moderate in 68 (31.4%) and 48 (24.9%), and low in 21 (9.6%) and 23 (12.4%), respectively (Table 3).

**Table 3:** Antiretroviral treatment adherence as assessed by different measures (pharmacy refill, self-reported questionnaire, and Visual Analogue Scale) and the composite adherence score at month 6 and 12 after ART initiation

Adherence variable	Adherence score	Adherence month 6	Adherence month 12
		*N* = 217	*N* = 185
Pharmacy refill	High	183 (84.3)	150 (81.1)
	Moderate	17 (7.8)	12 (6.5)
	Low	17 (7.8)	23 (12.4)
Self-reported questionnaire	High	159 (69.1)	143 (77.3)
	Moderate	43 (19.8)	31 (16.8)
	Low	15 (6.9)	11 (5.9)
Visual Analogue Scale (VAS)	High	150 (69.1)	135 (73.0)
	Moderate	39 (18.0)	25 (13.5)
	Low	28 (12.9)	25 (13.5)
Composite adherence score (CAS)	High	128 (59.0)	116 (62.7)
	Moderate	68 (31.4)	46 (24.9)
	Low	21 (9.6)	23 (12.4)

Data are in numbers and percentages [n (%)]

Differences within adherence estimates were further assessed by comparing correlation coefficients at each adherence time-point (Table 4). All adherence measures were correlated with each other. Significant correlation with the CAS by Spearman correlation were seen for all comparisons (*p* <  0.001), ranging from 0.36 to 0.83. However, the highest linear correlation was observed between the VAS and CAS which remained consistent both at 6 and 12 months with a Spearman correlation of 0.83.

**Table 4:** Correlation coefficient between each two adherence measures and between each adherence measure and the CAS at 6 and 12 months (Spearman correlation)

	Timing of adherence evaluation							
	6 month				12 month			
Adherence Tool	Pharmacy refill	Self-report	VAS	CAS	Pharmacy refill	Self-report	VAS	CAS
Pharmacy refill	1.00	0.42	0.52	0.62	1.00	0.36	0.66	0.72
Self-report	–	1.00	0.53	0.77	–	1.00	0.49	0.71
VAS	–	–	1.00	0.83	–	–	1.00	0.83

### Adherence and virological suppression

Of the 185 women assessed for adherence at months 12, VL was performed in 165 (90.7%) women. Of those 139 (84.2%) had undetectable VL < 40 copies/mL, 14 (8.5%) low level replication between 40 and 999 copies/mL, and 12 (7.3%) evidence for virological treatment failure ≥1000 copies/mL. Viral suppression < 1000 copies/mL was positively associated with high adherence as estimated by CAS in 100%, with moderate adherence in 89.5% and low adherence in 52.9% (Table 5). All adherence measures showed statistically significant differences between high adherence when compared with low adherence and virological suppression. In bivariate regression analysis there was a strong positive association between virological failure and low adherence for all adherence tools. Using the CAS, all clients who reported high adherence were virologically suppressed and moderate adherence showed statistically significant difference with virological suppression when compared to low adherence [OR 7.6, (95%CI, 1.8–30.8)] .

**Table 5:** Association between adherence scores by different adherence tools and virological suppression in pregnant and breastfeeding women at 12 months after ART initiation

Adherence variable	Participants who received VL assessments *N* = 165	VL ≥ 1000 copies/mL *N* = 12 (7.3)	VL < 1000 copies/mL *N* = 153 (92.7)	^a^OR (95%CI)	*P*-value
Pharmacy refill					
Low adherence	17 (10.3)	6/17 (35.3)	11/17 (64.7)	14.4 (3.8–54.8)	0.0001
Moderate adherence	11 (6.7)	1/11 (9.1)	10/11 (90.9)	2.6 (0.3–24.8)	0.395
High adherence	137 (83.0)	5/137 (3.6)	132/137 (96.4)	1	
Self-report					
Low adherence	7 (4.2)	4/7 (57.1)	3/7 (42.9)	21.2 (4.0–111.2)	0.0003
Moderate adherence	25 (15.2)	2/25 (8.0)	23/25 (92.0)	1.4 (0.3–6.9)	0.695
High adherence	133 (80.6)	6/133 (4.5)	127/133 (95.5)	1	
VAS					
Low adherence	18 (10.9)	9/18 (50.0)	9/18 (50.0)	124.0 (14.1–1090.4)	< 0.0001
Moderate adherence	22 (13.3)	2/22 (9.1)	20/22 (90.9)	12.4 (1.1–143.0)	0.06
High adherence	125 (75.8)	1/125 (0.8)	124/125 (99.2)	1	
CAS					
Low adherence	17 (10.3)	8/17 (47.1)	9/17 (52.9)	7.6 (1.8–30.8)	0.005
Moderate adherence	38 (23.0)	4/38 (10.5)	34/38 (89.5)	1	
High adherence	110 (66.7)	0 (0.0)	110/110 (100)	–	

Data are in numbers and percentages [n (%)].^a^binary logistic regression, OR (crude odd ratio)

### Risk factors associated with low adherence

In both bivariate and multivariate regression analyses adjusted for socio-demographics and other HIV and laboratory characteristics, low adherence was statistically significant with younger as compared to older ages [aOR 3.8, (95%CI, 1.4–10.6)], completed primary compared to completed secondary education and above [aOR 2.7, (95%CI, 1.4–5.2)] and employment in the informal sector when compared to unemployment [aOR 1.9, (95%C, 1.0–3.6)] (Table 6).

**Table 6:** Risk factors for low adherence using the composite adherence score (CAS) at 12 months following Option B+ initiation with adjusted odd ratios (aOR) for 5 health facilities in Kumba health district

Variables	Low adherence *N* = 85	Moderate or high adherence *N* = 162	Bivariate analysis^a^		Multivariate analysis^b^	
			OR (95% CI)	P-value	aOR (95% CI)	*P*-value
Age (years)						
15–24	29 (42.0)	40 (58.0)	2.4 (0.9,5.9)	0.07	3.8 (1.4, 10.6)	0.01
25–34	48 (33.3)	96 (66.7)	1.6 (0.7,3.9)	0.27	1.9 (0.8,4.6)	0.18
35 and above	8 (23.5)	26 (76.5)	1		1	
Educational Level						
None	1(20.0)	4 (80.0)	0.8 (0.1,7.8)	0.87	1.3 (0.1,15.2)	0.81
Completed primary	59 (44.0)	75 (56.0)	2.6 (1.5,4.6)	0.001	2.7 (1.4,5.2)	0.001
Completed secondary and above	25 (23.1)	83 (76.9)	1		1	
Occupation						
Unemployed	23 (24.2)	72 (58.8)	1		1	
Employed formal sector	5 (38.5)	8 (61.5)	2.0 (0.6,6.6)	0.28	3.9 (1.0,14.8)	0.055
Employed informal sector	57 (41.0)	82 (59.0)	2.2 (1.2,3.9)	0.01	1.9 (1.0,3.6)	0.05
Religious affiliation						
Catholic	20 (36.4)	35 (63.6)	1		1	
Protestants (Presbyterians/Baptist)	26 (28.3)	66 (71.7)	0.7 (0.3,1.4)	0.31	0.6 (0.3,1.4)	0.24
Pentecostals	39 (41.1)	56 (58.9)	1.2 (0.6,2.4)	0.57	1.1 (0.5,2.3)	0.88
Muslim	0 (0.0)	5 (100.0)	–		–	
CD4 at initiation						
< 350 cells/μL	41 (38.7)	65 (61.3)	1.4 (0.8,2.4)	0.22	1.4 (0.8,2.3)	0.23
> 350 cells/μL	44 (31.2)	97 (68.8)	1		1	

Data are in numbers and percentages [n (%)],^a^binary logistic regression, OR (crude odd ratio), aOR (adjusted odd ratio)]. ^**b**^adjusted for age, level of education, occupation, religious afiliation, WHO staging and CD4 count at ART initiation

Religious affiliation, marital status, initial CD4 T-cell count at ART initiation, WHO stage and timing of ART initiation were not associated with low adherence.

### Reasons for missed medication doses and factors that helped to remind medication taking

During adherence assessment at month 12, 121 women gave reasons why doses were missed. Frequently cited reasons were forgetfulness by 43 (35.5%), travel away from home by 21 (24.0%) and lack of transport to the clinic by 28 (23.1%) women. Side effects, stigmatization, distracted by the baby, being away for work and being involved in church or other social activities were less frequently cited. Asking about factors that helped to remind medications taking, 172 women provided one or more responses. The most frequently cited were the use of cell phone by 64 (37.2%) women, 63 (36.6%) indicated that drug taking had become a routine in their life so it occurs more as an instinct, and 22 (12.8%) mentioned the use of alarm clocks. Among the less frequently cited, 14 (8.1%) declared being reminded by their husbands, 4 (2.3%) relied on a television series and 4 (2.3%) had their drugs by their bedside. Two additional tables show the clients responses to these two questions in more details [see Additional file 1; Tables 1 and 2].

## Discussion

Using a composite adherence score (CAS) we determined adherence in association with viral suppression and risk factors of poor adherence in a cohort of HIV-positive pregnant and breastfeeding women who remained in care at 6 and 12 months after initiating ART as part of Option B+. The study demonstrated that of those women who remained in care after Option B+ initiation, 59.0 and 31.4% were either highly or moderately adhering to their treatment as per our CAS tool at 6 month, and 62.7 and 24.9% at month 12, respectively. The predictive accuracy of the CAS tool was reflected by virological suppression rates, which were 100% in women who scored high, 89.5% who scored moderate, and 52.9% who scored low with the CAS tool at month 12. Low CAS adherence as compared to moderate adherence was significantly associated with virological failure (OR 7.6, *p* = 0.005). In our cohort, moderate to high adherence scores by the CAS tool were sufficient to reach more than 90% of viral suppression (< 1000 copies/mL) as defined by the UNAIDS targets, thus 90.8% of women at 6 months and 87.6% at 12 months were considered with good adherence to reach durable treatment response. Our adherence levels were comparable with findings of earlier reports of good adherence with Option B+ from Eastern Africa [13, 32], but slightly higher than adherence reported for the same target group from an Option A study [33]. Adequate adherence assessment is important to better understand and optimise the success of the Option B+ programme.

A key strength of this study was the use of a CAS to assess adherence in this population. To our knowledge this is the first study to measure adherence to ART in the context of Option B+ using a CAS. We found a high level of correlation between all single adherence measures (pharmacy refill, self-reported adherence, and VAS) and the CAS which were also all associated with the 12 months viral suppression indicating the validity of each of the measures. It has been argued that the use of a CAS to measure adherence may be cumbersome in routine clinical settings [34]. Our observed close correspondence between the VAS and the CAS suggests that a single and less cumbersome tool like the VAS could be more conveniently used in clinical settings, and this observation was also reported in other older studies [30, 31, 35]. Furthermore, in a recent review of adherence and retention beyond Option B+ the authors’ claimed that the paucity of literature on adherence in this population was due to the lack of adherence measure systematically used in routine care [36]. This thus justifies the importance of a simple tool like the VAS for routine adherence assessment at the point of care. Among clients with virologic failure who reported a high adherence, 50% came from self-reported adherence indicating its high vulnerability to social desirability and recall biases [29]. Despite its mediocre performance as an adherence assessment tool in predicting virologic failure, we would still argue that self-reported questionnaires remains useful in providing additional information necessary to provide adequate adherence support to clients with adherence challenges.

Viral suppression rates in women who remained in care at 12 months in our study (92.7%) outreached the UNAIDS 2020 target [21], suggesting that viral suppression is achievable both in pregnancy and/or breastfeeding. However, 8.5% of our women had low replicating viraemia between 40 and 999 copies/mL, and given the low genetic resistance barrier of non-nucleotide reverse transcriptase inhibitor containing regimens, close monitoring is required to promptly identify treatment failure and switch clients to a second line therapy.

Risk factors for low adherence to ART were younger age, low level of education and employment in the informal sector. Younger age and low level of education had been reported as predictors of lost to follow-up and treatment discontinuation [8, 20]. Older women with more experience and better self-care skills/abilities may be more responsible towards their health compared to younger women [20]. Employees in the informal sector have their own unique work challenges and stresses, which can adversely affect their ability to concentrate on health and medication adherence. A South African study showed that conflict with work commitment and the difficulty of disclosing ones HIV status to an employer affected ARV adherence in women on Option B+ [37]. Potential strategies to mitigate these challenges include flexible clinics opening hours like evenings or weekends when most workers are free and do not need employer’s permission or a day off just to attend clinic.

Like other studies from sub-Saharan Africa, most participants in this study cited forgetfulness, being away from home and lack of transport to the health facility as possible reasons for missing doses [32, 38, 39]. Our study participants also indicated that reminder aids like cell phones, alarm clocks and reminder from family members helped improved their adherence. A good proportion of the respondents (36.6%) claimed that medication taking had become a routine for them so they needed no reminders. Of these, 88.2% had viral suppression showing that they had in fact adapted this routinely. This claim for those not virally suppressed may just covey their good intentions to continue taking their medication or methodological problems such as social desirability concerns. Recent studies have shown that the use of cell phones short message reminders can greatly improve adherence [40–42]. The role of family members like husbands and sisters to remind clients take their medication could improve adherence [13, 33].

### Limitations

The main limitation in this study was that adherence and virological suppression might had been overestimated as assessment was limited to women who retained in treatment, and may therefore not be representative of all HIV-positive women engaged in the Cameroon’s Option B+ programme. Despite the fact that we prefaced with a normalising language, the self-reported questionnaires and VAS may still had been prone to recall and social desirability biases. The small sample size also limited further analyses. Despite these limitations, our findings had shown that the VAS and the CAS both reliably predicted virological response in a clinical context thus could be used to assess adherence especially in resource limited settings where VL access may be limited. We therefore, believe that the study findings are useful to inform implementation of PMTCT Option B+ and to a certain extent the Test and Treat strategy in Cameroon and other comparable settings.

## Conclusions

During the first year of the Option B+ implementation in Cameroon we used a novel adherence tool, which effectively provided an adherence score that correlated well with viral suppression. However, younger, less educated and informal sector employed pregnant and breastfeeding women may need more attention for optimal adherence to help reduce the risk of virological failure.

## Additional file

Additional file 1: **Table S1.** Reasons for missing ART doses amongst women on Option B+ in Kumba health district. **Table S2.** Means of reminding women to take ART amongst women on Option B+ in Kumba Health district. During adherence assessment at month 12, women were asked about reasons why medication doses were missed, and 65.4% (121) provided one or more reasons. Frequently cited reasons were forgetfulness 35.5% (43), travel away from home 24.0% (29) and lack of transport to the clinic 23.1% (28). Stigmatization, being distracted by the baby, being away for work and being involved in church or other social activities were less frequently cited. Asking about means which women used to remind themselves of medications taking, 93.0% (172) provided one or more responses. The most frequently cited were the use of cell phone 37.2% (64), many indicated that medication taking had become a routine in their life so it occurs more as an instinct 36.6% (63), and 18.8% (22) mentioned the use of alarm clocks. Among the less frequently cited, 7.6% (13) declared being reminded by their husbands, 2.3% (4) relied on a TV series and 2.3% (4) others had their drugs by their bedside. (PDF 34 kb)

## Abbreviations

ANC: Antenatal care
aOR: Adjusted odds ratio
ART: Antiretroviral therapy
ARV: Antiretroviral
CAS: Composite adherence score
CBCHS: Cameroon Baptist Convention Health Service
CD4: Cluster of differentiation 4
CI: Confidence interval
CIH: Centre for International Health
DZIF: German Centre for Infection Research
HIV: Human Immunodeficiency Virus
HIV/AIDS: Human Immunodeficiency Virus/Acquired Immunodeficiency Syndrome
IWC: Infant welfare clinic
LMU: University of Munich
OR: Crude odds ratio
PEPFAR: President’s emergency plan for AIDS relief
PMTCT: Prevention of Mother-To-Child Transmission
UNAIDS: Joint United Nations Programme on HIV/AIDS
VAS: Visual analogue scale
VL: Viral load
WHO: World Health Organization

## References

[1] Mills EJ (2006). Adherence to antiretroviral therapy in sub-Saharan Africa and North America: a meta-analysis. Jama.

[2] Bangsberg DR, Preventing HIV (2008). Antiretroviral resistance through better monitoring of treatment adherence. J Infect Dis.

[3] Ngarina M (2015). Virologic and immunologic failure, drug resistance and mortality during the first 24 months postpartum among HIV-infected women initiated on antiretroviral therapy for life in the Mitra plus study, Dar Es Salaam, Tanzania. BMC Infect Dis.

[4] WHO. Consolidated guidelines on the use of antiretroviral drugs for treating and preventing HIV infection: summary of key features and recommendations. June. 2013:2013.

[5] WHO, Consolidated guidelines on the use of antiretroviral drugs for treating and preventing hiv infection: Recommendations for a Public Health approach. 2016. 27466667

[6] Takow SE (2013). Time for option B+? Prevalence and characteristics of HIV infection among attendees of 2 antenatal clinics in Buea, Cameroon. Journal of the International Association of Providers of AIDS Care.

[7] CDC. Impact of an innovative approach to prevent mother-to-child transmission of HIV--Malawi, July 2011–September 2012. MMWR Morb Mortal Wkly Rep. 2013, 62(8):148. PMC4604864 23446514

[8] Atanga PN (2016). Retention in care and reasons for discontinuation of lifelong antiretroviral therapy in a cohort of Cameroonian pregnant and breastfeeding HIV-positive women initiating ‘option B+‘in the south west region. Tropical Med Int Health.

[9] Coutsoudis A (2013). Is option B+ the best choice?: forum. Southern Afr J HIV Med.

[10] Haas AD (2016). Retention in care during the first 3 years of antiretroviral therapy for women in Malawi’s option B+ programme: an observational cohort study. Lancet HIV.

[11] Tenthani L (2014). Retention in care under universal antiretroviral therapy for HIV-infected pregnant and breastfeeding women ('Option B+') in Malawi. AIDS (London, England).

[12] Rollins NC (2014). Defining and analyzing retention-in-care among pregnant and breastfeeding HIV-infected women: unpacking the data to interpret and improve PMTCT outcomes. J Acquir Immune Defic Syndr.

[13] Ebuy H, Yebyo H, Alemayehu M (2015). Adherence level to and predictors of option B+ PMTCT program in Tigray, northern Ethiopia. Int J Infect Dis.

[14] Haas AD (2016). Adherence to antiretroviral therapy during and after pregnancy: cohort study on women receiving care in Malawi's option B+ program. Clin Infect Dis.

[15] Nieuwkerk PT, Oort FJ (2005). Self-reported adherence to antiretroviral therapy for HIV-1 infection and virologic treatment response: a meta-analysis. J Acquir Immune Defic Syndr.

[16] Kitahata MM (2004). Pharmacy-based assessment of adherence to HAART predicts virologic and immunologic treatment response and clinical progression to AIDS and death. Int J STD AIDS.

[17] Berg KM, Arnsten JH (2006). Practical and conceptual challenges in measuring antiretroviral adherence. J Acquir Immune Defic Syndr.

[18] Fokam J (2016). Short communication: population-based surveillance of HIV-1 drug resistance in Cameroonian adults initiating antiretroviral therapy according to the World Health Organization guidelines. AIDS Res Hum Retrovir.

[19] Mitiku I (2016). Factors associated with loss to follow-up among women in option B+ PMTCT programme in Northeast Ethiopia: a retrospective cohort study. J Int AIDS Soc.

[20] Tweya H (2014). Understanding factors, outcomes and reasons for loss to follow-up among women in option B+ PMTCT programme in Lilongwe, Malawi. Tropical Med Int Health.

[21] UNAIDS (2014). 90–90-90: an ambitious treatment target to help end the AIDS epidemic.

[22] Parienti J-J (2008). Not all missed doses are the same: sustained NNRTI treatment interruptions predict HIV rebound at low-to-moderate adherence levels. PLoS One.

[23] Kobin AB, Sheth NU (2011). Levels of adherence required for virologic suppression among newer antiretroviral medications. Ann Pharmacother.

[24] Bezabhe Woldesellassie M., Chalmers Leanne, Bereznicki Luke R., Peterson Gregory M. (2016). Adherence to Antiretroviral Therapy and Virologic Failure. Medicine.

[25] MOPH (2016). National Guidelines on Taskshifting for the Management of HIV/AIDS.

[26] CNLS, Directives nationales des prevention et de prise en charge du VIH au Cameroon*.* National Guidelines, 2015.

[27] Steel G, Nwokike J, Joshi MP (2007). Development of a multi-method tool to measure ART adherence in resource-constrained settings: the South Africa experience.

[28] Vinten G (1998). Taking the threat out of threatening questions. J R Soc Promot Heal.

[29] Wagner G, Miller LG (2004). Is the influence of social desirability on patients' self-reported adherence overrated?. J Acquir Immune Defic Syndr.

[30] Walsh JC, Mandalia S, Gazzard BG (2002). Responses to a 1 month self-report on adherence to antiretroviral therapy are consistent with electronic data and virological treatment outcome. Aids.

[31] Giordano TP (2004). Measuring adherence to antiretroviral therapy in a diverse population using a visual analogue scale. HIV Clin Trials.

[32] Ayuo P (2013). Frequency and factors associated with adherence to and completion of combination antiretroviral therapy for prevention of mother to child transmission in western Kenya. J Int AIDS Soc.

[33] Igwegbe A, Ugboaja J, Nwajiaku L (2010). Prevalence and determinants of non-adherence to antiretroviral therapy among HIV-positive pregnant women in Nnewi, Nigeria. Int J Med Med Sci.

[34] Simoni JM (2006). Self-report measures of antiretroviral therapy adherence: a review with recommendations for HIV research and clinical management. AIDS Behav.

[35] Oyugi JH (2004). Multiple validated measures of adherence indicate high levels of adherence to generic HIV antiretroviral therapy in a resource-limited setting. J Acquir Immune Defic Syndr.

[36] Myer L (2017). Beyond “option B+”: understanding antiretroviral therapy (ART) adherence, retention in care and engagement in ART Services among pregnant and postpartum women initiating therapy in sub-Saharan Africa. J Acquir Immune Defic Syndr.

[37] Clouse K (2014). “What they wanted was to give birth; nothing else”: barriers to retention in option B+ HIV care among postpartum women in South Africa. J Acquir Immune Defic Syndr.

[38] Shubber Z (2016). Patient-reported barriers to adherence to antiretroviral therapy: a systematic review and meta-analysis. PLoS Med.

[39] Kim MH (2016). Why did I stop? Barriers and facilitators to uptake and adherence to ART in option B+ HIV care in Lilongwe, Malawi. PLoS One.

[40] Fenerty SD (2012). The effect of reminder systems on patients’ adherence to treatment. Patient Prefer Adherence.

[41] Hardy H (2011). Randomized controlled trial of a personalized cellular phone reminder system to enhance adherence to antiretroviral therapy. AIDS Patient Care STDs.

[42] Mbuagbaw L, Bonono-Momnougui RC, Thabane L (2012). Considerations in using text messages to improve adherence to highly active antiretroviral therapy: a qualitative study among clients in Yaounde, Cameroon. HIV/AIDS (Auckl).

