# Supplementary material for: Using a composite adherence tool to assess ART response and risk factors of poor adherence in pregnant and breastfeeding HIV-positive Cameroonian women at 6 and 12 months after initiating option B+
Source: BMC Pregnancy Childbirth. 2018 Oct 25;18:418. doi: 10.1186/s12884-018-2058-9 (PMC6202832; doi:10.1186/s12884-018-2058-9)
Supplement: Supplementary file 1 — Table S1. Reasons for missing ART doses amongst women on Option B+ in Kumba health district. Table S2. Means of reminding women to take ART amongst women on Option B+ in Kumba Health district. During adherence assessment at month 12, women were asked about reasons why medication doses were missed, and 65.4% (121) provided one or more reasons. Frequently cited reasons were forgetfulness 35.5% (43), travel away from home 24.0% (29) and lack of transport to the clinic 23.1% (28). Stigmatization, being distracted by the baby, being away for work and being involved in church or other social activities were less frequently cited. Asking about means which women used to remind themselves of medications taking, 93.0% (172) provided one or more responses. The most frequently cited were the use of cell phone 37.2% (64), many indicated that medication taking had become a routine in their life so it occurs more as an instinct 36.6% (63), and 18.8% (22) mentioned the use of alarm clocks. Among the less frequently cited, 7.6% (13) declared being reminded by their husbands, 2.3% (4) relied on a TV series and 2.3% (4) others had their drugs by their bedside. (PDF 34 kb) [file 12884_2018_2058_MOESM1_ESM.pdf]

Table 1

Reasons for missing ART doses amongst women on Option B+ in Kumba health district.

**Reasons for potentially not taking medications**	**N**	**% Respondents**
**Total women**	**185**	
**Non respondents**	**64**	**34.6**
**Respondents**	**121**	**65.4**
– Forgetfulness	43	35.5
– Travel away from home	29	24.0
– Lack of transport to come to pick up ARV	28	23.1
– Side effects mainly dizziness	6	5.0
– Away for work	5	4.1
– Baby distraction	5	4.1
– Stigmatisation	5	4.1
– Involved in church or social activities	4	3.3
– Lack of food	2	1.7
– Child Vaccination is over	1	0.8

NB* *Percentatges are out of those who responded to each question. Some women gave more than one reason so total resposese may add up to over total respndents and same for percentatges (>100%).*

Table 2

Means of reminding women to take ART amongst women on Option B+ in Kumba Health district.

**Treatment reminder**	**N**	**% Respondents**
**Total women**	**185**	
**Non respondents**	**13**	**7.0**
**Respondents**	**172**	**93.0**
– Phone alarm	64	37.2
– Become a daily routine	63	36.6
– Clock alarm	22	12.8
– Husband	14	8.1
– Television series	4	2.3
– Drugs by my bedside	4	2.3
– Sister	1	0.6
– Church bell	1	0.6

NB* *Percentatges are out of those who responded to each question. Some women gave more than one reason so total resposese may add up to over total respndents and same for percentatges (>100%).*
